# Activation of 5-HT4 receptors reverses stress-induced dopamine system dysregulation

**DOI:** 10.1016/j.neuropharm.2025.110810

**Published:** 2025-12-18

**Authors:** Olivia J. Yang, Stephanie M. Perez, Daniel J. Lodge

**Affiliations:** a Department of Pharmacology and Center for Biomedical Neuroscience, University of Texas Health Science Center, San Antonio, TX, 78229, USA; b South Texas Veterans Health Care System, Audie L. Murphy Division, San Antonio, USA

## Abstract

Stress can profoundly impact brain function, particularly in circuits regulating dopamine transmission. Increased mesolimbic dopamine activity is a well-documented consequence of stress exposure, contributing to maladaptive behavioral and cognitive outcomes. Previous studies have identified a multisynaptic circuit that modulates dopamine neuron population activity in the ventral tegmental area (VTA), highlighting potential intervention points for mitigating stress-induced dopamine dysregulation. One such target is the 5-hydroxytryptamine-4 receptor (5-HT4R), which is expressed in key brain regions involved in dopamine system regulation, making it a promising candidate for pharmacological intervention. Here, we demonstrate that the 5-HT4R agonist BIMU8 effectively restores normal dopamine system function following stress exposure without altering baseline dopamine population activity in control male rats. Interestingly, in female rats, BIMU8 increased dopamine neuron population activity specifically during proestrus and estrus, suggesting that estrogen may play a role in serotoninergic modulation of mesolimbic dopamine function. Intracranial administration of BIMU8 into multiple brain regions indicates that its effects may be mediated through modulation of activity in the nucleus accumbens (NAc). These findings highlight 5-HT4R activation as a potential strategy for normalizing stress-induced alterations in dopamine system function.

## 1. Introduction

Stress is a significant environmental factor that can profoundly alter brain function, particularly in circuits regulating motivation, reward processing, and cognitive flexibility (McManus et al., 2022; McEwen, 2017; Jett et al., 2017). One of the key consequences of stress exposure is dysregulation of the dopamine system, which plays a crucial role in psychiatric disorders (Baik, 2020). Chronic or acute stress have been shown to alter dopamine neuron population activity in the ventral tegmental area (VTA), a change associated with maladaptive behavioral outcomes (McCoy et al., 2022; Elam et al., 2021; Valenti et al., 2011). Understanding the mechanisms underlying stress-induced alterations in dopamine function is critical for identifying therapeutic strategies to mitigate its impact.

Dopamine dysregulation has been extensively studied in the context of psychiatric disorders. The dopamine hypothesis of psychosis, for example, posits that increased mesolimbic dopamine activity contributes to the emergence of symptoms of hallucinations and delusions (Howes et al., 2009, 2012). This theory is supported by findings of increased markers of striatal dopamine transmission in patients with psychosis compared to healthy controls (Howes et al., 2009, 2012). Specifically, a recent meta-analysis of schizophrenia patients demonstrated consistent increases in markers of presynaptic dopamine bioavailability in striatal regions (Howes et al., 2012). In rodent models, these changes have been linked specifically to an increase in dopamine neuron population activity rather than alterations in firing rate or burst firing (Perez et al., 2022a). Dopamine neuron activity states (i.e. population activity, firing rate, burst firing) are controlled by distinct afferent pathways (Floresco et al., 2003). Specifically, population activity is regulated by convergent glutamatergic inputs from the ventral hippocampus (vHipp), and paraventricular nucleus of the thalamus (PVT) to the nucleus accumbens (NAc) which reduces activity in the ventral pallidum (VP) to disinhibit dopamine neuron activity in the VTA (Perez and Lodge, 2018; Lodge and Grace, 2007) (Fig. 3A). We have previously shown that rats exposed to acute stress will exhibit selective increases in dopamine neuron population activity and no changes in firing rate or burst firing of dopamine neurons (McCoy et al., 2022; Elam et al., 2021). Thus, targeting brain regions in this circuit may be a more effective point of intervention to treat psychosis.

One potential target for mitigating stress-induced dopamine dysregulation is the Gs-protein coupled 5-hydroxytryptamine-4 receptor (5-HT4R), which is expressed in several key regions involved in regulating mesolimbic dopamine transmission, including the vHipp, VP, and NAc (Vilaró et al., 2005; Licht et al., 2010). The 5-HT4R plays a role in cognition, and activation of these receptors can improve memory and increase functional connectivity in both humans and rodents (de Cates et al., 2021, 2023; Lamirault and Simon, 2001; Restivo et al., 2008). Genetic inactivation of 5-HT4R in mice causes depressive-like phenotypes, and 5-HT4R activation elicits antidepressant-like and anxiolytic-like effects in rodents (Lucas et al., 2001, 2007). Interestingly, 5-HT4R activation is also prophylactic against stress, reducing vulnerability to stress in mouse models (Chen et al., 2020). However, the effects of 5-HT4R activation on stress-induced aberrant dopamine system function have yet to be characterized.

In this study, we utilized a two-day inescapable foot shock paradigm to induce stress-related increases in VTA dopamine neuron population activity. Administration of the selective 5-HT4R agonist BIMU8 effectively reversed these stress-induced alterations, as well as those observed in another rodent model characterized by dopamine system hyperactivity (the MAM model). Intracranial administration of BIMU8 into different brain regions further revealed that the effects of 5-HT4R activation on dopamine system function may be mediated through receptors in the NAc. These findings suggest that targeting 5-HT4Rs may represent a novel strategy for normalizing dopamine system function following stress exposure.

## 2. Materials and methods

All experiments were performed in accordance with the guidelines outlined in the USPH Guide for the Care and Use of Laboratory Animals and were approved by the Institutional Animal Care and the Use Committees of UT Health San Antonio and the US Department of Veterans Affairs.

### 2.1. Animals

Studies involving two-day inescapable foot shock stress were performed on untreated, adult male and female Sprague Dawley (SD) rats (250–600 g; Envigo; Indianapolis, IN, USA) that were group housed. Multiple litters of adult (>12 weeks) male and female methylazoxymethanol acetate (MAM)-treated rats were generated as previously described (Lodge, 2013; Moore et al., 2006). All rats were kept in a temperature-controlled environment, on a 12 h light/dark cycle, with *ad libitum* access to food and water. Estrus cycle was examined in female rats by vaginal cytology as previously reported (Marcondes et al., 2002; Perez et al., 2019).

### 2.2. Drug administration

For systemic administration, BIMU8 (1, 3, 10 mg/kg, *i.p.*; K_i_ 33.9 ± 8.0 nM) or vehicle (saline), were administered 15 min prior to electrophysiology. For intracranial administration, a 26-gauge canula (Plastics One; Roanoke County, VA, USA) was lowered into either the vHipp (AP −5.3 mm, ML ± 5.0 mm from bregma, DV −7.0 mm ventral of the brain surface), VP (AP −0.0 mm, ML ± 2.3 mm from bregma, DV −8.0 mm ventral of the brain surface), or NAc (AP +1.4 mm, ML ± 1.3 mm from bregma, DV −7.6 mm ventral of the brain surface). An internal canula (Plastics One), extending 1 mm past the end of the guide cannula was used to deliver a single injection of either BIMU8 (0.75 μg/0.75 μL) or vehicle (saline, 0.75 μL) 15 min prior to electrophysiology. The 5-HT4R antagonist, SB-204070 (0.75 μg/0.75 μL) was delivered to the NAc 15 min prior to systemic BIMU8 in a subset of rats. Our dosing strategy was guided by the systemic efficacy range and adjusted for local diffusion volume within the target region. At the cessation of all electrophysiological recording, rats were rapidly decapitated.

### 2.3. Two-day inescapable foot shock stress

Rats were randomly assigned to control or stress groups and placed in a 30.5 × 25.4 × 30.5 cm^3^ square conditioning chamber with a stainless-steel grid floor (Coulbourn Instruments, Whitehall, PA, USA). Stressed rats received a two-day inescapable foot shock treatment where they were exposed to 60 × 15 s 0.8 mA foot shocks with an average inter-trial interval of 30 s and 25 % standard deviation (±7.5 s) for approximately 40 min. Control rats were placed in the conditioning chamber but did not receive foot shocks. Electrophysiology experiments were conducted 24–48 h following stress.

### 2.4. In vivo extracellular dopamine neuron recordings

**Fig. 1.**
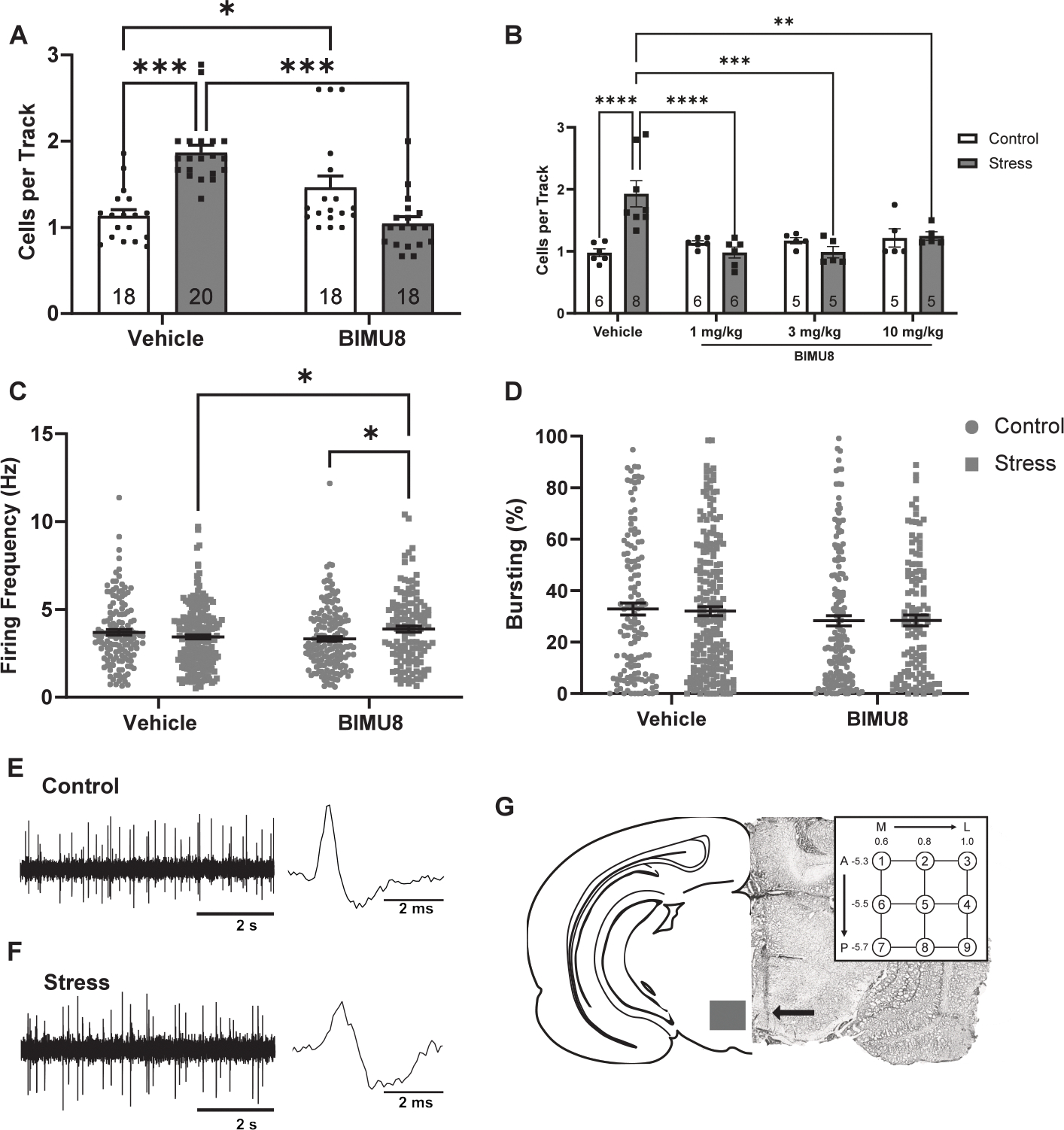
Systemic administration of BIMU8, a 5-HT4R agonist, reverses aberrant dopamine function elicited by stress. **(A)** Population activity (average number of spontaneously active dopamine neurons per electrode track) is significantly increased in stressed rats compared to controls (Holm-Sidak; *t* = 5.568, *p* < 0.001), which is reversed with BIMU8 (Holm-Sidak; *t* = 6.233, *p* < 0.001). Two-way ANOVA; *F*_(1,70)Treatment_ = 6.795, *p* = 0.011; *F*_(1,70)Interaction_ = 37.138, *p* < 0.001. BIMU8 increases dopamine neuron population activity in control animals (Holm-Sidak; *t* = 2.435, *p* = 0.017) **(B)** BIMU8 decreases dopamine neuron population activity in stressed male rats compared to vehicle at 1 mg/kg (Holm-Sidak; *t*= 5.685, *p* < 0.001), 3 mg/kg (**(C)** BIMU8 produces increase in dopamine neuron firing rate in stressed rats compared to vehicle-treated stressed rats (Holm-Sidak; *t* = 2.168, *p* = 0.031) and BIMU8-treated control rats (Holm-Sidak; *t* = 2.607, *p* = 0.009) Two-way ANOVA; *F*_(1,640)Interaction_ = 7.866, *p* = 0.005. **(D)** No differences in percent burst firing were detected. Two-way ANOVA; *F*_(1,640)Treatment_ = 3.925, *p* = 0.048 **(E**–**F)** Representative dopamine recording and action potential traces from **(E)** control and **(F)** stressed animals. **(G)** Representative brain slice with an electrode track (arrow) through the ventral tegmental area. Recording electrodes are lowered in a predetermined grid pattern of vertical tracks separated by 0.2 mm (inset). Data represented as mean ± SEM; *p < 0.05 **p < 0.005 ***p < 0.001.

**Fig. 2.**
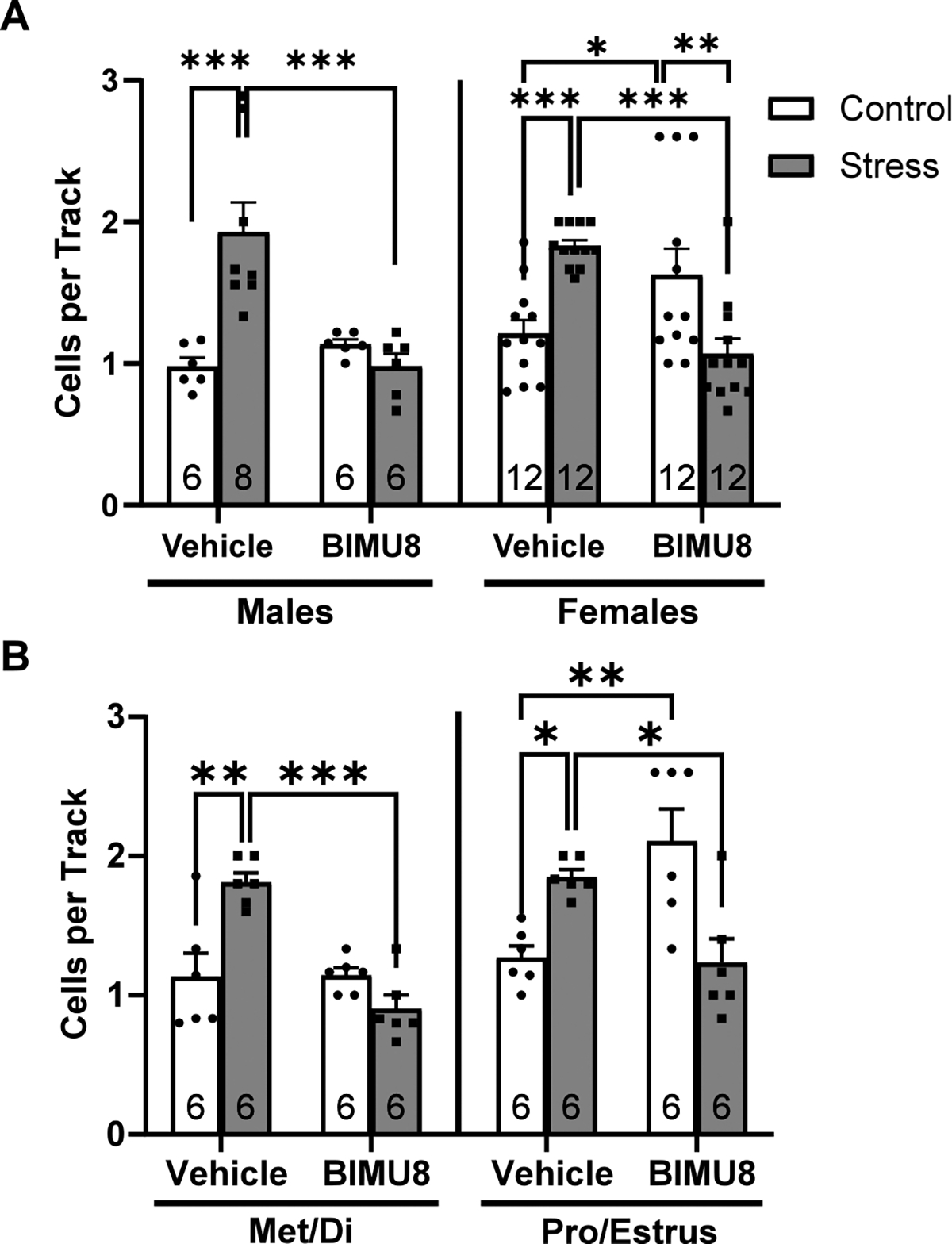
BIMU8 increases dopamine neuron population activity in control female rats in pro/estrus. **(A)** Population activity stratified by sex. In males, stress increased dopamine neuron population activity (Holm-Sidak; *t*= 4.877, *p* < 0.001) and BIMU8 reversed stress-induced increases (Holm-Sidak; *t*= 4.856, *p* < 0.001). Two-way ANOVA; *F*_(1,25)Strain_ = 7.760, *p* = 0.011; *F*_(1,25)Treatment_ = 7.606, *p* = 0.011; *F*_(1,25)Interaction_ = 15.034, *p* < 0.001. In females, stress increased dopamine neuron population activity (Holm-Sidak; *t*= 3.701, *p* < 0.001) and BIMU8 reversed stress-induced increases (Holm-Sidak; *t*= 4.551, *p* < 0.001). BIMU8 increased population activity in control females compared to vehicle (Holm-Sidak; *t*= 2.484, *p* = 0.017) and compared to BIMU8-treated stressed females (Holm-Sidak; *t*= 3.333, *p* = 0.002). Two-way ANOVA; *F*_(1,47)Interaction_ = 24.744, *p* < 0.001. **(B)** Population activity in females further stratified by cycle. In females in met/diestrus, stress increased dopamine neuron population activity (Holm-Sidak; *t* = 4.518, *p* < 0.001) and BIMU8 reversed stress-induced increases (Holm-Sidak; t = 6.039, p < 0.001). Two-way ANOVA; *F*_(1,20)Treatment_ = 17.793, p < 0.001; *F*_(1,20)Interaction_ = 18.687, *p* < 0.001. In females in pro/estrus, stress increased dopamine neuron population activity (Holm-Sidak; *t* = 2.575, *p* = 0.018) and BIMU8 reversed stress-induced increases (Holm-Sidak; *t* = 2.834, *p* = 0.01). BIMU8 increased population activity in control females compared to vehicle (Holm-Sidak; *t* = 3.768, *p* = 0.001). Two-way ANOVA; *F*_(1,20)Interaction_ = 21.793, *p* < 0.001. Data represented as mean ± SEM; *p < 0.05 **p < 0.005 ***p < 0.001.

Rats were anesthetized with 8 % chloral hydrate (400 mg/kg, *i.p.*) and placed in a stereotaxic apparatus. Supplemental anesthesia was administered to maintain suppression of limb compression withdrawal reflex. Extracellular glass microelectrodes (impedance ~6–10 MΩ) were lowered into the VTA (AP −5.3 mm, ML ± 0.6 mm from bregma, and DV −6.5–9.0 mm ventral of the brain surface) and spontaneously active dopamine neurons identified using previously established electrophysiological criteria (Ungless and Grace, 2012; Grace and Bunney, 1983). Briefly, dopamine neuron action potentials were characterized as biphasic action potentials with duration >2.0 ms and firing rate between 0.5 and 15 Hz. Electrode was lowered to make 6–9 vertical passes through the VTA separated by 200 μm. Three parameters of dopamine activity were measured and analyzed: the number of dopamine neurons firing spontaneously (population activity)(Lodge and Grace, 2011), basal firing rate, and burst firing (defined previously (Grace and Bunney, 1983)). Population activity was measured by dividing the number of spontaneously firing dopamine neurons by the number of vertical passes through the VTA to get an average number of “cells per track”. Analysis of dopamine neuron activity was performed using commercially available computer software (LabChart version 8; ADInstruments, Colorado Springs, CO, USA). Representative traces from dopamine neurons electrophysiological recordings are displayed in Fig. 1E–F and Fig. 2B–C.

### 2.5. RT-PCR

Rats were deeply anesthetized with 8 % chloral hydrate (400 mg/kg, *i.p.*) and rapidly decapitated 24 h after stress. The vHipp, VP, and NAc were dissected by tissue punch, submersed in RNA later, and stored in −80°C until processed. RNA was precipitated and separated by filtration using a commercially available kit (RNAqueous^™^ Total RNA Isolation Kit). RNA was converted to cDNA by a Reverse Transcription Kit (Applied Biosystems^™^). Real-time PCR was performed with FAM-labeled TaqMan primers targeting either 5-hydroxytryptamine (serotonin) receptor 4 (Rn00563402_m1) or glyceraldehyde-3-phosphate dehydrogenase (Rn01775763_gl). Detection of FAM-labeled DNA was performed by using a CFX384 Real-Time PCR Detection System (BioRad Laboratories; Hercules, CA, USA) and analyzed by BioRad’s CFX Manager software.

### 2.6. Histological verification

**Fig. 3.**
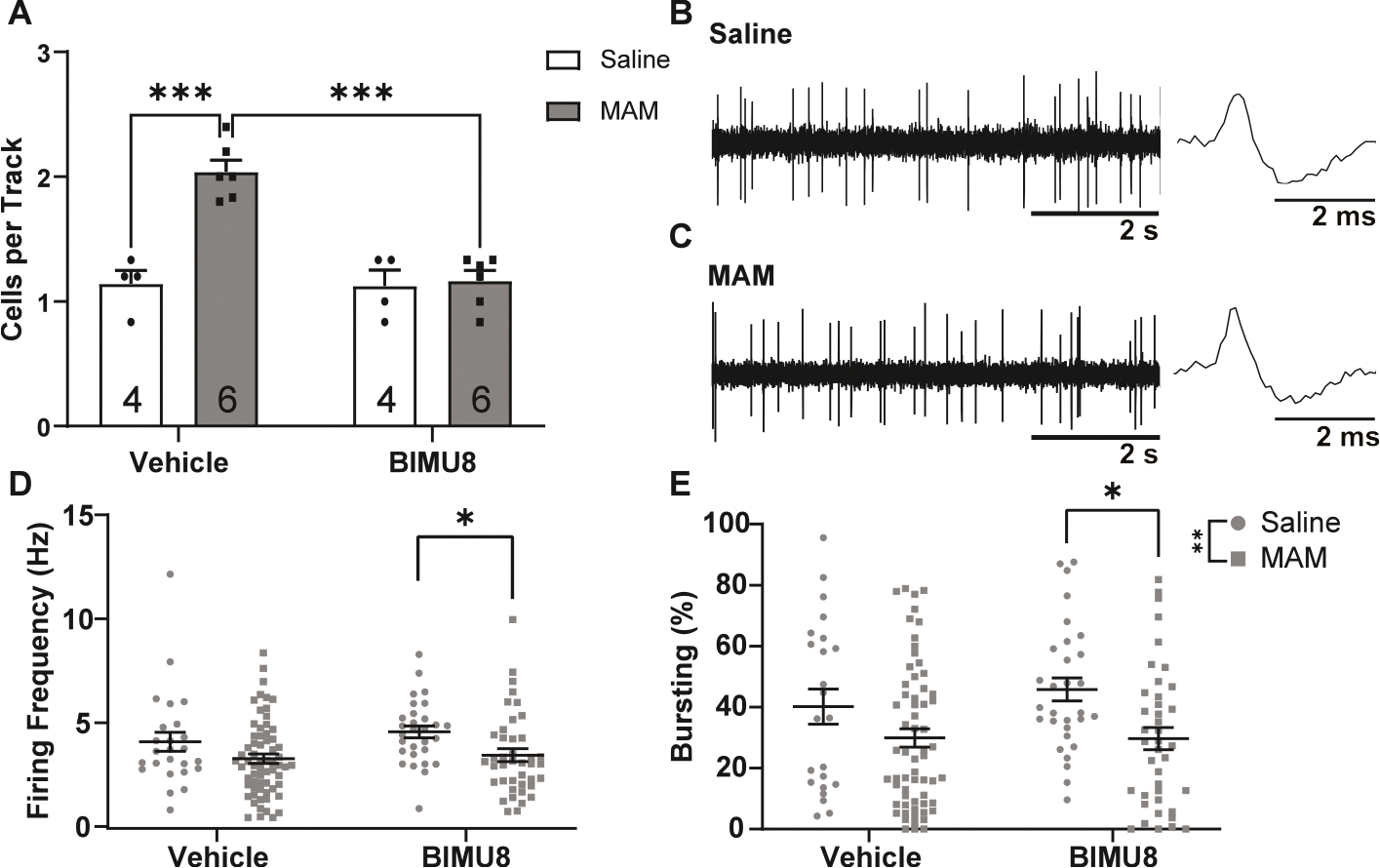
Systemic administration of BIMU8 restores normal dopamine neuron population activity in MAM rats. **(A)** population activity is significantly increased in MAM-treated rats compared to saline-treated rats (Holm-Sidak; *t* = 6.238, *p* < 0.001). Giving BIMU8 (1 mg/kg) to control animals does not affect population activity (Holm-Sidak; *t* = 0.273, *p* = 0.788), while giving BIMU8 to MAM rats significantly decreases population activity (Holm-Sidak; *t* = 6.798, *p* < 0.001). Two-way ANOVA; *F*_(1,16)Strain_ = 21.195, *p* < 0.001; *F*_(1,16)Treatment_ = 19.196, *p* < 0.001; *F*_(1,16)Interaction_ = 17.787, *p* < 0.001 **(B**–**C)** Representative dopamine recording and action potential traces from **(B)** saline-treated and **(C)** MAM-treated animals. **(D)** BIMU8 decreased firing rate in MAM rats compared to saline rats (Holm-Sidak; *t* = 2.822, *p* = 0.005) Two-way ANOVA; *F*_(1,151)Strain_ = 9.106, *p* = 0.003. **(E)** BIMU8 decreased bursting activity in MAM rats compared to saline rats. Two-way ANOVA; *F*_(1,151)Strain_ = 10.575, *p* = 0.001. Data represented as mean ± SEM; *p < 0.05 **p < 0.005 ***p < 0.001.

Electrode and cannula placement was confirmed by histological verification. Rats were rapidly decapitated at the completion of all electrophysiological experiments. Brains were extracted, fixed for at least 24 h (4 % formaldehyde in saline), and cryoprotected (10 % [wt/vol] sucrose in phosphate-buffered saline) until saturated. Brains were coronally sectioned (25 μm) using a cryostat (Leica). Sections containing electrode tracks were mounted onto gelatin-chrome alum-coated slides, stained with neutral red (0.1 %) and thionin acetate (0.01 %) and cover slipped with DPX mountant. Histological confirmation was performed of the recording electrode (VTA; Fig. 1G) or cannula (vHipp, VP, NAc; Fig. 3B) with reference to a stereotaxic atlas (Paxinos and Watson, 2006).

### 2.7. Statistical analysis

Electrophysiological analysis of dopamine neuron activity was performed using commercially available computer software (Lab Chart version 8; ADInstruments Ltd.; Chalgrove, Oxfordshire, UK). Data are represented as the mean ± SEM with *n* values representing the number of rats per experimental group, unless otherwise stated. Data were analyzed by t-test or two-way ANOVA (stress x drug or MAM x drug) and the Holm-Sidak post-hoc test was used when significant interactions were determined. Statistics were calculated using SigmaPlot (Systat Software Inc.; Chicago, IL, USA) or Prism software (GraphPad Software Inc., San Diego CA, USA) with significance determined at p < 0.05, and graphed with Prism software.

### 2.8. Materials

BIMU8 (4374) was purchased from R&D Systems (Minneapolis, MN, USA). SB-204070 (1373) was purchased from Tocris (Bristol, UK). Chloral hydrate (C8383) and DPX mountant (06522) were sourced from Sigma-Aldrich (St. Louis, MO, USA). RNAqueous^®^ Total RNA Isolation Kit (AM1912), TaqMan^®^ primers for 5-hydroxytryptamine (serotonin) receptor 4 (Rn00563402_m1) or glyceraldehyde-3-phosphate dehydrogenase (Rn01775763_gl), and the High-Capacity cDNA Reverse Transcription Kit (4368814) were acquired from Thermo Fisher Scientific (Mico, TX, USA). All other chemicals and reagents were of either analytical or laboratory grade and purchased from standard suppliers.

## 3. Results

### 3.1. Systemic administration of BIMU8, a 5-HT4R agonist, reverses aberrant dopamine function elicited by stress

Consistent with previous reports, control rats displayed an average of 1.13 ± 0.10 cells per track (*n* = 18 rats) and this was significantly increased in rats that underwent two-day inescapable foot shock stress (*n* = 20 rats; 1.87 ± 0.09 cells per track; Fig. 1A)(McCoy et al., 2022; Elam et al., 2021). Importantly, systemic administration of the 5-HT_4_ receptor agonist BIMU8 normalized this stress-induced increase (n = 18 rats, 1.05 ± 0.10 cells per track), suggesting a potential therapeutic effect of 5-HT_4_R activation in restoring dopamine system homeostasis. Additionally, BIMU8 at increasing doses (3, 10 mg/kg) was also able to restore dopamine system function in stressed male rats (1 mg/kg: *n*= 6 rats, 0.98 ± 0.09 cells per track; 3 mg/kg: *n*= 5 rats, 0.99 ± 0.09 cells per track; 10 mg/kg: *n*= 5 rats, 1.25 ± 0.07 cells per track; Fig. 1B) without affecting controls (1 mg/kg: *n*= 6 rats, 1.14 ± 0.03 cells per track; 3 mg/kg: *n*= 5 rats, 1.17 ± 0.05 cells per track; 10 mg/kg: *n*= 5 rats, 1.22 ± 0.15 cells per track).

We also examined two additional parameters of dopamine neuron activity: average firing rate and percentage bursting. Vehicle-treated control rats displayed an average firing rate of 3.70 ± 0.16 Hz (*n* = 134 neurons; Fig. 1C), vehicle-treated stress rats displayed an average firing rate of 3.44 ± 0.12 Hz (*n* = 223 neurons). In comparison, BIMU8 produced a slight but significant increase in firing rate in stressed rats (*n* = 121 neurons, 3.88 ± 0.16 Hz) and control rats (*n* = 166 neurons, 3.32 ± 0.14 Hz). We observed a main effect of treatment on percent burst firing (Fig. 1D); however, we did not observe any significant differences between individual groups (control/vehicle: *n* = 134, 32.87 ± 2.21 %; stress/vehicle: *n* = 223, 32.01 ± 1.7 %; control/BIMU8: *n* = 166, 28.30 ± 2.0 %; stress/BIMU8: *n* = 121, 28.38 ± 2.32 %). This suggests that while BIMU8 influences dopamine neuron firing rate modestly, its primary effect lies in regulating the number of active dopamine neurons, a key determinant of tonic dopamine output.

Interestingly, we observed a significant increase in dopamine neuron population activity of control rats following systemic BIMU8 administration (*n* = 18 rats, 1.46 ± 0.10 cells per track). To explore this effect, we stratified the data by sex and found that this increase occurred only in females (Fig. 2A). When further stratified by estrus cycle, we found that the observed increase was driven by females in proestrus (pro) or estrus (est), as systemic BIMU8 produced a significant increase in VTA population activity specifically in this subset (*n* = 6 rats, 2.11 ± 0.23 cells per track; Fig. 2B when compared to vehicle-treated control females in pro/est (*n* = 6 rats, 1.29 ± 0.10 cells per track). However, BIMU8 had no effect in control females in metestrus (met) or diestrus (di) (*n* = 6 rats, 1.14 ± 0.05 cells per track) when compared to vehicle-treated control females in met/di (*n* = 6 rats, 1.13 ± 0.17 cells per track). These findings suggest that hormonal fluctuations across the estrus cycle may modulate the dopaminergic effects of 5-HT_4_R activation.

Stressed females displayed significant increases in population activity compared to controls regardless of being in pro/est (*n* = 6 rats, 1.850 ± 0.05 cells per track) or met/di (*n* = 6 rats, 1.81 ± 0.10 cells per track). Importantly, BIMU8 still reversed stress-induced increases in population activity for females in pro/est (*n* = 6 rats, 1.23 ± 0.17 cells per track) and met/di (*n* = 6 rats; 0.91 ± 0.09 cells per track). These results support the conclusion that while baseline sensitivity to 5-HT_4_R activation may vary with hormonal status, the therapeutic effect of BIMU8 in reversing stress-induced dopamine system hyperactivity is preserved across the estrus cycle.

### 3.2. Systemic administration of BIMU8 restores normal dopamine neuron population activity in MAM rats

To determine whether the therapeutic effects of 5-HT_4_R activation generalize to other models of dopamine dysregulation relevant to psychiatric illness, we tested BIMU8 in the methylazoxymethanol acetate (MAM) model of schizophrenia. This neurodevelopmental model, in which pregnant dams receive MAM treatment at gestational day 17, produces circuit-level alterations that mimic several aspects of schizophrenia, including elevated dopamine neuron population activity and impaired hippocampal function (Lodge, 2013; Moore et al., 2006). Consistent with previous studies (Lodge, 2013; Perez et al., 2022b), rats treated with MAM (*n* = 6 rats, 2.04 ± 0.09 cells per track) displayed an increased number of spontaneously active dopamine neurons compared to rats treated with saline (*n* = 4, 1.14 ± 0.11 cells per track; Fig. 3A). Systemic administration of BIMU8 restored dopamine population activity back to baseline in MAM rats (*n* = 6 rats, 1.16 ± 0.09 cells per track) without altering population activity in saline controls (*n* = 4 rats, 1.13 ± 0.11 cells per track). These data indicate that BIMU8 is capable of restoring normal dopamine system function not only following acute stress, but also in a developmental model of chronic circuit dysfunction. This broad efficacy supports the potential of 5-HT_4_R activation as a therapeutic strategy for disorders characterized by dopamine system dysregulation.

MAM rats displayed small but significant decreases in average firing rate (MAM/vehicle: *n* = 62 neurons, 3.36 ± 0.24 Hz; MAM/BIMU8: *n* = 40 neurons, 3.53 ± 0.30 Hz; Fig. 3D) and percent burst firing (MAM/vehicle: *n* = 62 neurons, 29.90 ± 3.00 % bursting; MAM/BIMU8: *n* = 40 neurons, 29.68 ± 3.74 % bursting; Fig. 3E) compared to saline-treated rats (saline/vehicle: *n* = 23 neurons, 4.17 ± 0.38 Hz; 40.20 ± 4.93 % bursting; saline/BIMU8: *n* = 29 neurons, 4.65 ± 0.35 Hz; 45.81 ± 4.32 % bursting). BIMU8 significantly decreased firing rate and percent burst firing in MAM rats compared to saline rats receiving BIMU8.

### 3.3. Intracranial injections of BIMU8 into NAc reverses stress-induced alterations in dopamine system function

**Fig. 4.**
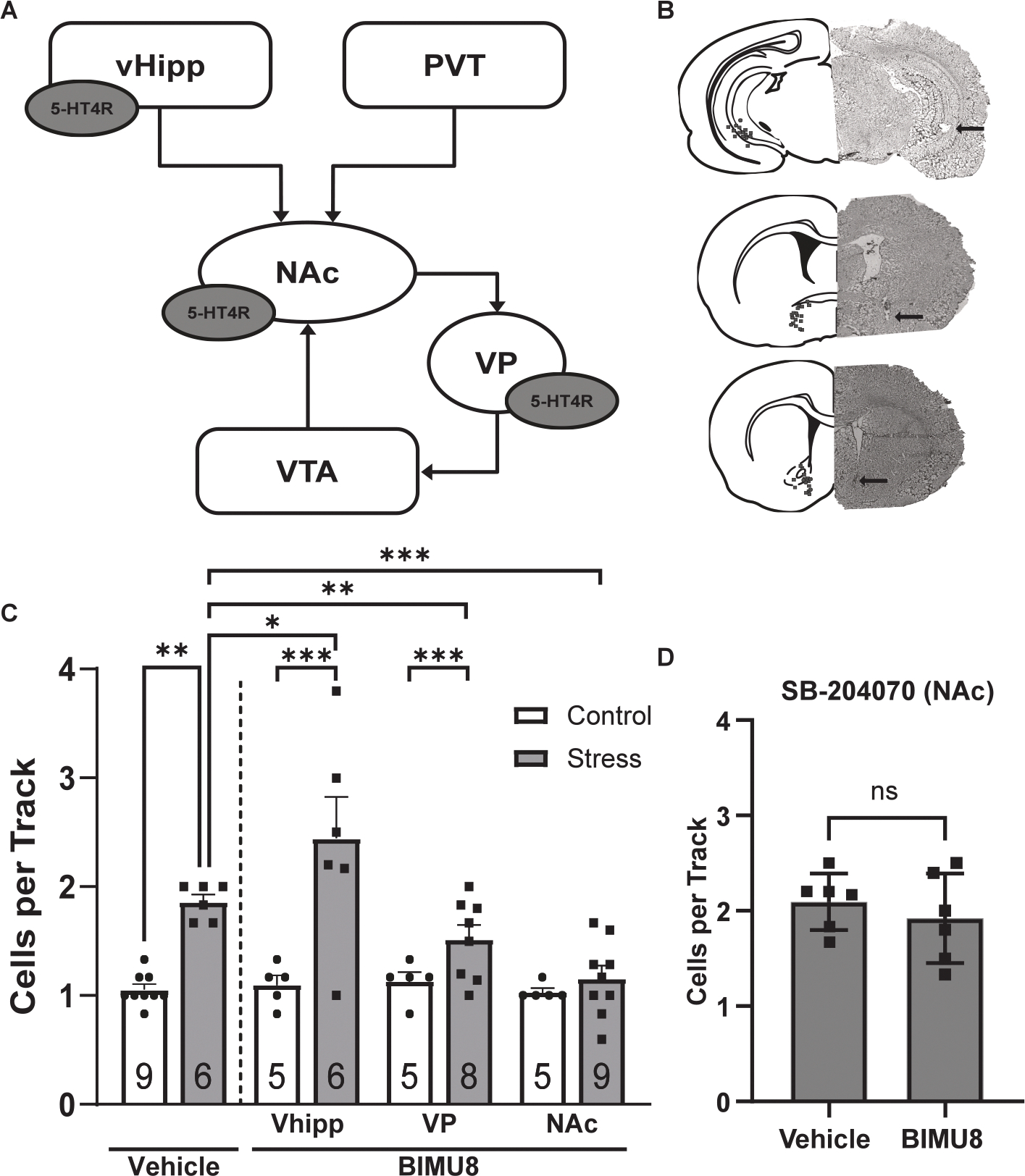
Intracranial injections of BIMU8 into NAc reverses stress-induced alterations in dopamine system function. **(A)** Schematic indicating the multisynaptic circuit regulating VTA dopamine neuron population activity and brain regions with 5-HT4R expression. **(B)** representative images (right) of cannula tracks (arrow) in vHipp (top), VP (middle) and, NAc (bottom) with corresponding schematic of the brain section (left) and each injection site represented by grey circles for vehicle and grey squares for BIMU8. **(C)** Population activity in control and stressed animals following intracranial injections of vehicle or BIMU8 into the vHipp, VP, and NAc. Stress increased dopamine neuron population activity in vehicle-treated animals (Holm-Sidak; *t* = 6.677, *p* < 0.001). Vehicle-treated control rats were not significantly different from control rats receiving intra-vHipp (Holm-Sidak; *t*= 0.174, *p*= 0.864), intra-VP (Holm-Sidak; *t*= 0.590, *p*= 0.560), or intra-NAc (Holm-Sidak; *t*= 0.179, *p*= 0.860) injections of BIMU8. Intra-vHipp injections of BIMU8 in stressed rats (Two-way ANOVA, *F*_*(1,26)Strain_vHipp*_ = 36.091, *p* < 0.001) elicited an increase in VTA dopamine population activity compared to vehicle-treated stressed rats (Holm-Sidak; *t* = 2.209, *p* = 0.037). A significant decrease was observed in stressed rats receiving intra-VP delivery of BIMU8 (Two-way ANOVA, *F*_*(1,28)Strain_VP*_ = 45.324, *p* < 0.001) compared to vehicle (Holm-Sidak; *t* = 2.970, *p* = 0.006). However, BIMU8 treatment into the VP was not sufficient to return population activity back to baseline. Microinjections of BIMU8 into the NAc of stressed rats (Two-way ANOVA, *F*_*(1,29)Strain_NAc*_ = 32.237, p < 0.001, *F*_*(1,29)Treatment_NAc*_ = 19.663, p < 0.001, *F*_*(1,29)Interaction_NAc*_ = 17.382, p < 0.001) significantly decreased population activity compared to vehicle-treated stressed rats (Holm-Sidak; *t* = 6.417, *p* < 0.001). **(D)** Giving the 5-HT4R antagonist, SB-204070 into the NAc blocked systemic BIMU8 from reversing elevations in dopamine population activity in stressed rats (*t*-test, *t* = 0.7574, *p* = 0.4663). Data represented as mean ± SEM; *p < 0.05 **p < 0.005 ***p < 0.001.

To determine the brain region mediating the effects of BIMU8, we administered intracranial injections of BIMU8 into various brain regions known to express 5-HT4R as well as regulate mesolimbic dopamine activity (Fig. 4A) (Perez and Lodge, 2018; Vilaró et al., 2005). To minimize the number of rats being used, we combined vehicle-treated groups between regions, as there were no observable differences within the controls. Furthermore, we restricted our analysis to males and females in met/di to avoid potential variability due to hormonal status. Control rats receiving vehicle displayed an average of 1.06 ± 0.08 cells per track (*n* = 9 rats), and stressed rats receiving vehicle exhibited a significant increase in the average number of cells per track (*n* = 6 rats, 1.86 ± 0.09 cells per track; Fig. 3C).

In control animals, intra-vHipp (*n*= 5 rats, 1.10 ± 0.15 cells per track), intra-VP (*n*= 5 rats, 1.13 ± 0.08 cells per track), or intra-NAc (*n*= 5 rats, 1.03 ± 0.10 cells per track) injections of BIMU8 had no effect on dopamine neuron population activity, indicating that local 5-HT4R activation alone is not sufficient to increase dopamine activity in the absence of stress. In contrast, intra-vHipp injections of BIMU8 in stressed rats (*n* = 6 rats, 2.44 ± 0.18 cells per track) elicited an increase in VTA dopamine population activity compared to vehicle-treated stressed rats (Fig. 4C). A modest but significant decrease was observed in stressed rats receiving intra-VP delivery of BIMU8 (*n* = 8 rats, 1.52 ± 0.08 cells per track) compared to vehicle. However, BIMU8 treatment into the VP was not sufficient to return population activity back to baseline.

The most robust reversal was observed with intra-NAc BIMU8 in stressed rats (n = 9), which significantly reduced population activity (1.16 ± 0.08 cells per track) to near-control levels. These findings indicate that activation of 5-HT4Rs in the NAc is sufficient to normalize dopamine system function following stress. To validate that the systemic effects of BIMU8 are mediated by activity at 5-HT4R in the NAc, we administered the selective 5-HT4R antagonist, SB-204070, into the NAc prior to systemic BIMU8 or vehicle in stressed rats. While SB-204070 alone did not influence population activity in vehicle-treated rats (*n* = 6 rats, 2.09 ± 0.12 cells per track), local infusion of the antagonist prevented systemic BIMU8 from reversing stress-induced increases in dopamine system function (*n* = 6 rats, 1.92 ± 0.19 cells per track; Fig. 4D). These data confirm that 5-HT4R activation in the NAc is both necessary and sufficient to mediate the normalization of dopamine population activity following stress. Together, these results identify the NAc as a key site of action for 5-HT4R-mediated modulation of the dopamine system in this model.

### 3.4. htr4 mRNA expression is not altered by foot shock stress

**Fig. 5.**
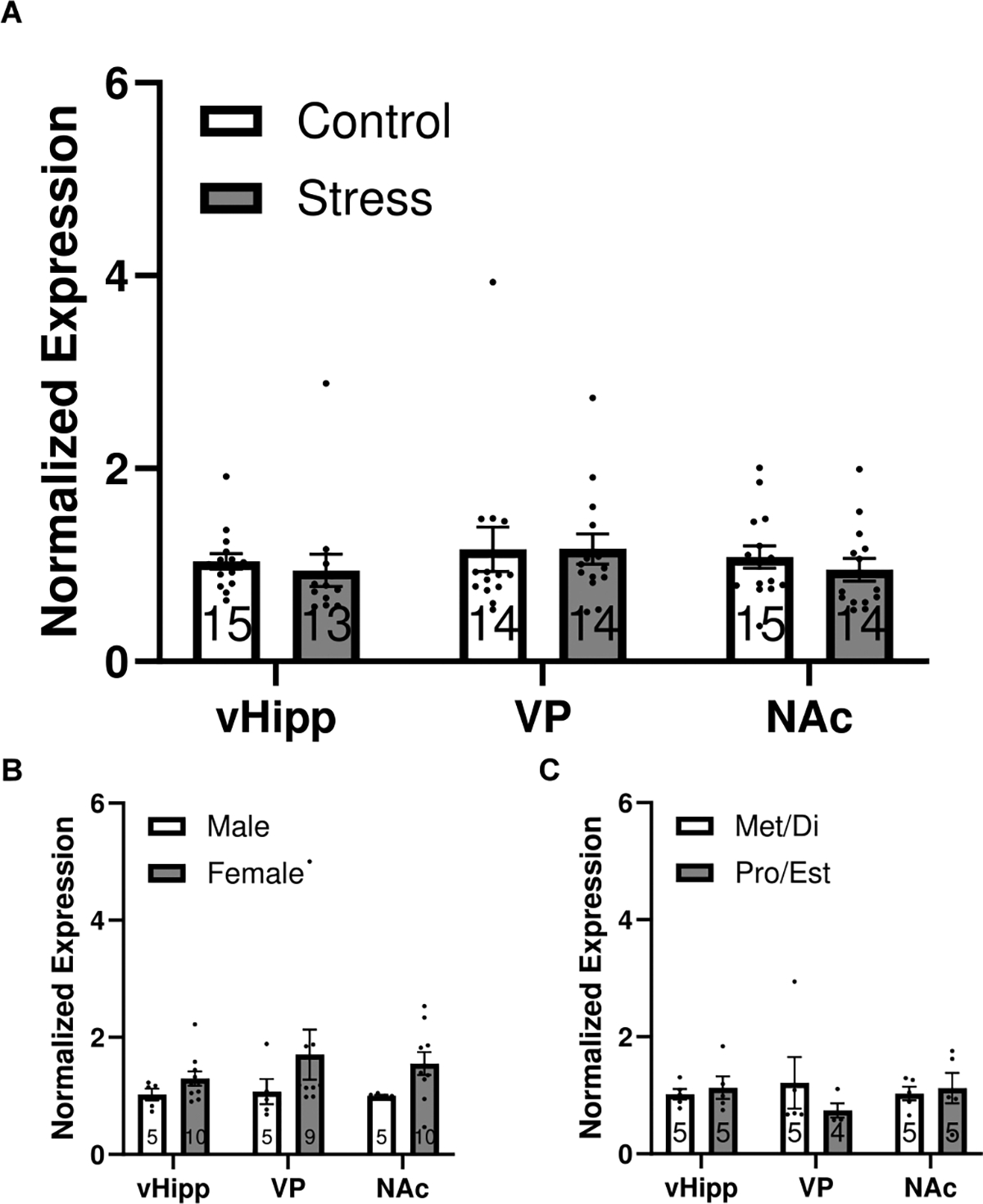
htr4 mRNA expression does not change with stress. **(A)** mRNA expression of *htr4* in the vHipp (*t*-test, *t* = 0.5158, *p* = 0.6103), VP (*t*-test, *t* = 0.0167, *p* = 0.9868), and NAc (*t*-test, *t* = 0.8061, *p* = 0.4272) normalized to control animals. No significant differences were detected between groups. **(B)** *htr4* mRNA expression is not changed between control male and control females in any brain region. **(C)** *htr4* mRNA is not changed with estrus cycle in control females in any brain region. All data was normalized using Lidak method to control **(A)**, male **(B)**, or met/di **(C)** for each brain region. Data represented as mean ± SEM.

We performed RT-PCR on tissue taken from the vHipp, VP, and NAc of control and stressed rats to evaluate transcription of the 5-HT4R-encoding gene, *htr4*. Across all regions, *htr4* transcript levels were not significantly affected by stress (Control/vHipp: *n* = 15, 1.04 ± 0.08 c.f.; Stress/vHipp: *n* = 13, 0.95 ± 0.17 c.f.; Control/VP: *n* = 14, 1.16 ± 0.22 c.f.; Stress/VP: *n* = 14, 1.17 ± 0.16 c.f.; Control NAc: *n* = 15, 1.08 ± 0.12 c.f.; Stress/NAc: *n* = 14, 0.95 ± 0.12 c.f.; Fig. 5A), indicating that stress does not alter *htr4* transcription in these areas. To assess whether sex or hormonal state might influence htr4 expression under baseline conditions, we further compared transcript levels between control males and females (Fig. 5B) as well as between control females in metestrus/diestrus versus proestrus/estrus (Fig. 5C). No significant differences were observed in either comparison, suggesting that htr4 expression is stable across sex and estrous cycle stages. These findings indicate that the ability of BIMU8 to normalize dopamine system activity following stress is not mediated by stress-induced changes in htr4 expression. Rather, the behavioral and physiological effects of BIMU8 likely reflect changes in downstream circuit function or receptor sensitivity, rather than transcriptional regulation of 5-HT4Rs.

## 4. Discussion

Increased dopamine neurotransmission is a well-documented consequence of stress and is hypothesized to underlie certain psychiatric disorders (Baik, 2020; Almuqrin et al., 2023; Mizrahi et al., 2012). In this study, we utilized a two-day inescapable foot shock paradigm to induce stress-related increases in VTA dopamine population activity and demonstrated that the 5-HT4R agonist BIMU8 effectively reversed this dysregulation, likely through its effects in the NAc. These findings suggest a potential therapeutic approach for mitigating stress-induced alterations in dopamine system function.

Rodent models cannot fully recapitulate complex psychiatric conditions; however, they provide valuable insight into the neural circuits underlying stress-induced dopamine dysregulation. We have previously shown that foot shock stress increases dopamine neuron population activity and produces deficits in pre-pulse inhibition of the startle reflex and increased locomotor response to stimulants, behavioral phenotypes that are relevant to human stress-related disorders (McCoy et al., 2022; Elam et al., 2021), supporting its use as a model for investigating circuit-level mechanisms involved in stress-induced dopamine system dysregulation.

It should be noted that other stress paradigms can induce different responses in the dopamine neurons. For example, repeated social defeat, a predominantly psychological stressor, has been shown to increase firing rate and burst firing, reflecting engagement of circuits that regulate these parameters. Furthermore, other stressors (i.e. restraint stress, chronic mild stress, and chronic cold exposure) can result in sustained reductions in dopamine neuron population activity (Valenti et al., 2011; Chang and Grace, 2013, 2014; Moore et al., 2001) that more closely align with stress-induced depressive-like behaviors (Chang and Grace, 2014; Belujon and Grace, 2017). This difference is likely due to the recruitment of distinct neural circuits based on the intensity and duration of the stressor. For example, repeated stressors such as footshock or restraint reliably increase dopamine neuron population activity in the VTA (Elam et al., 2021; Valenti et al., 2011, 2012), which can be normalized by inhibiting vHipp (McCoy et al., 2022; Elam et al., 2025), suggesting that vHipp plays a key role in stress-induced enhancement of dopamine system function. In contrast, chronic and milder stressors, such as chronic mild stress, have been shown to decrease dopamine neuron population activity, and this reduction can be reversed by attenuating basolateral amygdala (BLA) activity (Chang and Grace, 2013, 2014). It is also plausible that the neural substrates mediating stress effects on dopamine activity shift over time. Early increases in dopamine activity may be driven by hippocampal inputs, while delayed or chronic suppression could emerge from amygdala-related processes (Grace, 2016). Thus, the acute hyperdopaminergic response observed here may reflect an early phase of stress adaptation, with the potential for later compensatory decreases in dopamine activity depending on the persistence of stress-induced circuit changes. Longitudinal studies would be needed to test this temporal dynamic more directly. Furthermore, repeated social defeat, a predominantly psychological stressor, has been shown to increase firing rate and burst firing, reflecting engagement of circuits that regulate these parameters.

Using the foot shock model, we demonstrate that BIMU8 can reverse stress-induced increases in VTA dopamine activity. Studies have shown that 5-HT4Rs play a role in modulating nigrostriatal dopaminergic function, and giving a 5-HT4R antagonist can prevent the increases in firing rate of nigrostriatal dopamine neurons facilitated by haloperidol or morphine (Lucas et al., 2001; Porras et al., 2002). However, less is known about the relationship between 5-HT4Rs and mesolimbic dopamine functions. While control animals treated with BIMU8 displayed increased dopamine population activity compared to control animals treated with vehicle, this increase was driven by rats in pro/est of the estrous cycle. During proestrus, estrogen levels reach their peak, and likely play a role in the differential effects of BIMU8. Estrogen receptors are hormone-dependent nuclear receptors that act as transcription factors, and there are several estrogen-responsive genes. Because estrogen can act as a transcription factor, and estradiol induces expression of 5-HT4R mRNA in the anterior pituitary (Papageorgiou and Denef, 2007), we compared *htr4* expression throughout the estrus cycle in female control and stressed rats. We hypothesized that estrogen may be altering htr4 transcription; however, we did not observe significant differences in mRNA in any of the brain regions between pro/est and met/di females, indicating that the observed results cannot be attributed to transcription-level alterations in 5-HT4R expression. While this finding does not change the overall interpretation of this study, given that BIMU8 normalized population activity in stressed female rats irrespective of cycle, subsequent studies that investigate the link between estrous cycle and serotoninergic control of mesolimbic dopamine transmission are warranted.

Also important to note is the changes in firing rate and bursting activity of dopamine neurons observed in this study. Dopamine neurons exhibit three different activity states, that may change based on afferent inputs. For example, manipulations of pedunculopontine tegmental nucleus can affect burst firing of dopamine neurons without altering firing rate or population activity (Floresco et al., 2003). Similarly, manipulations of ventral subiculum in the ventral hippocampus affects population activity without altering burst firing or firing rate (Floresco et al., 2003). Specifically, the subicular control over population activity engages the circuitry described in our study (Floresco et al., 2003). Our particular stress paradigm, which only affects dopamine neuron population activity, thus seems to selectively engage this circuit, without affecting other afferent regulations of dopamine neuron activity states. Indeed, aversive and stressful stimuli such as foot/tail shock and pinch can have profound effects on dopamine neuron firing rate (Marinelli and McCutcheon, 2014). However, these effects are often transient, and occur on a time-scale immediate to the aversive event. Changes in firing rate at the time point we perform electrophysiology (24 h after stress) have not been observed. Based on our hypothesis that BIMU8 engages a population activity-specific circuit, we did not expect changes in firing rate or burst firing with BIMU8 administration. However, under pathological conditions, 5-HT4R activation may modulate inputs to dopamine neurons, resulting in subtle changes in firing rate or recruitment patterns.

To determine whether these findings could translate to other rodent models that display alterations in dopamine system function related to psychosis, we performed the same experiments in MAM rats. MAM, when given to rats at gestational day 17, leads to hyperactivity within the vHipp and increases dopamine population activity in the VTA via projections through the NAc and VP (Lodge, 2013; Lodge and Grace, 2011). Interestingly, BIMU8 also normalized VTA dopamine population activity in MAM rats without affecting control animals. One limitation of the study is we only included male MAM rats in the study. While the inclusion of females would have provided useful information regarding estrous cycle regulation of dopamine activity, the primary objective was to demonstrate that BIMU8 normalizes dopamine system function across distinct models of dopamine dysregulation.

Because BIMU8 was able to reverse elevated dopamine activity in both the MAM and stressed rats, it is likely that BIMU8 is targeting one of these brain regions that modulate dopamine neuron population activity. Thus, we delivered BIMU8 intracranially into the brain regions known to express 5-HT4R and modulate VTA dopamine activity. We found that BIMU8 in vHipp increased dopamine population activity. In the hippocampus, 5-HT4R mRNA is found on glutamatergic cells (Vilaró et al., 2005; Peñas-Cazorla and Vilaró, 2015). These cells project to NAc, and when activated, increase VTA dopamine population activity (Perez and Lodge, 2018). The 5HT4R activates the Gs-AC-cAMP signaling pathway and activation of pyramidal cells in the vHipp is most likely causing the BIMU8-induced increase in dopamine activity. While functional evidence indicates 5-HT4R modulation of GABAergic signaling within hippocampal CA1 (Lecouflet et al., 2021), the precise cell-type attribution (e.g., to specific hippocampal interneuron subpopulations) remains to be fully established. Interestingly, this effect was not observed in control animals, suggesting that stress sensitizes the hippocampus, allowing BIMU8 to potentiate its influence on the dopamine system. Under non-stressed conditions, the hippocampus is relatively quiescent, and BIMU8 alone may not be sufficient to activate this circuit and drive downstream dopamine system changes. In contrast, BIMU8 delivered into the VP significantly decreased population activity in stressed rats; however, this attenuation did not reach baseline, indicating only a partial contribution of VP to the systemic effect of BIMU8.

BIMU8 infusion into the NAc completely reversed stress-induced increases in dopamine neuron population activity, implicating this region as a key site of action for its therapeutic effects. Based on established circuitry, heightened NAc output from medium spiny neurons (MSN) can increase VTA dopamine activity by enhancing GABAergic inhibition of VP, which in turn disinhibits VTA dopamine neurons. Our findings suggest that 5-HT4R activation in the NAc may suppress this maladaptive NAc output, thereby restoring inhibitory control over the dopamine system. Although *htr4* mRNA has been detected on MSNs (Vilaró et al., 2005; Rebholz et al., 2018; Jean et al., 2012; Compan et al., 1996), previous literature and our data point toward a role for 5-HT4Rs on local inhibitory interneurons within the NAc. Specifically, activation of these interneurons could reduce MSN activity and dampen NAc output. This proposed mechanism aligns with prior reports of *htr4* expression in both GABAergic and parvalbumin-positive neurons in the basal forebrain (Peñas-Cazorla and Vilaró, 2015), and supports a model in which 5-HT4R agonism restores dopamine system function via modulation of local inhibitory microcircuits in the NAc.

The data detailed above suggest that 5-HT4R activation may be a novel therapeutic mechanism to treat stress-induced dopamine system dysfunction observed in patients with psychosis. Indeed, administration of 5-HT4R partial or full agonists is prophylactic against stress-induced depressive and anxiety-like behavior (Chen et al., 2020). Mice treated with corticosterone (CORT) and receiving chronic administration of the partial 5-HT4R agonist, RS-67,333 exhibit increased open-arm entries in elevated plus maze and decreased latency to approach food in novelty suppressed feeding task compared to vehicle treated CORT mice (Chen et al., 2020). Additionally, a single prophylactic injection of various 5-HT4R agonists (RS-67,333, prucalopride, PF-04995274) attenuates learned fear during 3-shock contextual fear conditioning (Chen et al., 2020). These findings highlight the potential of 5-HT4R agonists in mitigating stress-induced neurophysiological and behavioral deficits. Though the primary objective of the current study was to determine whether 5-HT4R activation could restore dopamine neuron population activity following stress to provide a physiological foundation for subsequent behavioral investigations, future work will directly evaluate how 5-HT4R activation modulates behavioral consequences of stress, linking the observed physiological normalization to functional outcomes. Indeed, a recent study utilizing electronic health records reported a reduced incidence of psychiatric disorders among patients taking prucalopride, an FDA-approved 5-HT4R agonist, compared to controls (de Cates et al., 2024). Notably, the most significant association was observed with psychotic disorders, although this was a secondary finding with very low incidence and warrants additional studies with a larger sample size. Given its established safety profile, future research may explore prucalopride’s effects on stress-induced dopamine system dysfunction to evaluate its potential for repurposing in the treatment of psychotic disorders.

Overall, our results provide compelling evidence that 5-HT4R activation represents a novel approach for modulating stress-induced dopamine dysfunction. Given its ability to normalize dopamine signaling without broadly suppressing dopamine transmission, targeting 5-HT4R may offer a promising strategy for developing safer and more effective treatments for stress-related conditions.

## Data Availability

All data supporting this article will be shared on reasonable request to the corresponding author.
